# The crossroads of Leber hereditary optic neuropathy and autosomal dominant optic Atrophy: Clinical profiles of patients with coexisting pathogenic genetic variants

**DOI:** 10.1016/j.ajoc.2025.102346

**Published:** 2025-04-29

**Authors:** Mohammed A. Halawani, Nooran O. Badeeb

**Affiliations:** a Department of Ophthalmology, King Khalid Eye Specialist Hospital, Riyadh, Saudi Arabia; b Department of Surgery, Ophthalmology Devision, College of medicine, University of Jeddah, Saudi Arabia; c Department of Ophthalmology, King Fahad Armed Forces Hospital, Ministry of Defense Health Services, Jeddah, Saudi Arabia

**Keywords:** Leber hereditary optic neuropathy (LHON), Autosomal dominant optic atrophy (ADOA), Vision loss, Pathogenic variants

## Abstract

**Purpose:**

Leber Hereditary Optic Neuropathy (LHON) and Autosomal Dominant Optic Atrophy (ADOA) are hereditary optic neuropathies characterized by mitochondrial dysfunctions causing destruction to the retinal ganglion cells and their axons, painless bilateral vision loss and symmetrical temporal pallor of the optic nerve. We present six intrafamilial cases with different manifestations of LHON and/or ADOA and their genetic variant profiles.

**Observations:**

Two brothers and their father had symptomatic bilateral vision loss, two sisters were asymptomatic, and the mother had left eye vision loss due to solar retinopathy; accompanied with headaches. Five of the patients had normal anterior and posterior segment exam aside from the affected optic nerves. The family pedigree showed an unaffected first generation and an affected male in the second generation. In the third generation, an affected male (the father in this family), diagnosed with optic atrophy due to *OPA1* c.2383C > T variant, married a woman (the mother) carrying the LHON *MT-ND4* m.11778G > A variant. Their offspring were one unaffected daughter, one affected daughter and two affected sons harboring both LHON and ADOA pathogenic variants inherited from their parents.

**Conclusion and importance:**

Mitochondrial optic neuropathies, which result in loss of retinal ganglion cells, are a substantial cause of visual impairment. Herein, we report two cases of combined LHON- and ADOA-causing pathogenic variants in two brothers, in addition to the genetic and ophthalmologic profile of their parents and two sisters.

## 1 Introduction

Hereditary optic neuropathies are a group of diseases with highly heterogeneous genetic and clinical characteristics and a minimum prevalence of one in 10,000.^1^ These disorders include Leber hereditary optic neuropathy (LHON, OMIM 535000) and autosomal dominant optic atrophy (ADOA, OMIM 165500), caused by mutations in mitochondrial DNA (mtDNA) or nuclear genes, yielding mitochondrial dysfunctions that induce damage to the retinal ganglion cells (RGC) and their axons, resulting in painless bilateral vision loss and symmetrical temporal pallor of the optic nerve.^2^, ^3^, ^4^

LHON is a maternally inherited mitochondrial illness that can cause acute or subacute bilateral vision loss. The prevalence of LHON is estimated at around 1 in 31,000–50,000 in the general population^5^, ^6^, ^7^ and at about 1 in 14,000 in adult men (significantly higher than in women).^7^ The majority of cases (90–95 %) are attributed to mtDNA pathogenic variants at positions m.11778G > A m.3460G > A, or m.14484T > C, affecting respiratory chain complex I nicotinamide adenine dinucleotide hydrogen (NADH) dehydrogenases (ND4, ND1, and ND6, respectively).^8^^,^^9^ Other gene alterations, environmental factors, and genetic penetrance contribute to LHON severity.^10^, ^11^, ^12^ Clinically, LHON is characterized by the sequential and painless onset of dense ceco-central scotomas with dyschromatopsia. Visual acuity at 6/60 or less, involvement of the fellow eye within the first few months to one year, visual field defects in the central field, severely affected color vision, but relatively well-preserved pupillary light responses.^8^ Some visual improvement is possible, particularly in patients carrying the 14,484 variant, with a 37–71 % chance of spontaneous partial visual recovery, compared to 4 % for other variants.^13^ Fundus examination at onset frequently shows thickening of the retinal nerve fiber layer (RNFL), hyperemia, peri-papillary telangiectasia, and mild tortuosity. The tissue used for sequence analysis is an important consideration in LHON molecular diagnosis, and total DNA should be isolated from different tissues for optimal LHON molecular diagnostics.^14^ The gold standard is skeletal muscle biopsy (invasive technique) and urine sediments.^15^

Autosomal DOA (ADOA) typically presents earlier than LHON, in the first decade of life. Prevalence ranges from 1:10,000 to 1:50,000, with the highest incidence in Denmark.^16^, ^17^, ^18^ ADOA penetrance is around 70 %, but depending on families, genetic variants and study criteria,^18^^,^^19^ it can vary from 43 %^20^ to 100 %.^21^ Vision loss is bilateral, simultaneous, gradual, and very slowly progressive over decades. Patients commonly exhibit reduced visual acuity, ceco-central scotomas, and blue/yellow dyschromatopsia. Most patients retain visual acuity of 20/200 or better, with one-third retaining 20/60 or better.^20^ The optic disc characteristically exhibits temporal pallor or excavation, which can progress to diffuse pallor.^22^^,^^23^ About 57–75 % of ADOA patients carry a variant in the optic atrophy type 1 (*OPA1*) gene, 1 % of patients a variant in the *OPA3* gene, and the rest variants in *OPA4*, *OPA5* or *OPA8*.^1^^,^^24^ Genetic deficiency of *OPA1* is associated with mitochondrial fragmentation and impaired respiratory capacity, as reported in primary cell cultures of human fibroblasts and murine RGCs.^25^^,^^26^ *OPA1* mutants exhibit preferential atrophy of high energy consumption glutamatergic synapses, found in highly metabolically active RGCs,^27^ while OFF-center RGCs with GABAergic synapses, which have a lower metabolic demand, are relatively unaffected.^28^^,^^29^

Here, we report the cases of two brothers and their sister, with bilateral optic atrophy caused by the co-occurrence of two rare genetic variants, resulting in coexisting LHON and ADOA. We also report on the clinical profile of the parents and an asymptomatic sister.

## 2 Case description

### 2.1 Patient consent

Consent to publish this case series has been obtained from the patients in writing.

### Ethical statement

The publication of this case series was approved by the Institutional Review Board at the King Fahd Armed Forces in Jeddah (Reference number REC 745, on November 11, 2024).

### 2.2 Methodology of the genetic test

Peripheral blood was withdrawn and was sent for CentoXome® Solo analysis by a CLIA-certified laboratory in Germany (CENTOGENE GmbH). Genomic DNA was extracted from nucleated blood cells and then enzymatically fragmented. Target regions were enriched using DNA capture probes. These regions include approximately 41 Mb of the human coding exome (targeting >98 % of the coding RefSeq from the human genome build GRCh37/hg19), as well as the mitochondrial genome. The generated library was sequenced on an Illumina platform to obtain at least 20x coverage depth for >98 % of the targeted bases. An in-house bioinformatics pipeline, including read alignment to GRCh37/hg19 genome assembly and revised Cambridge Reference Sequence (rCRS) of the Human Mitochondrial DNA (NC_012920), variant calling, annotation, and comprehensive variant filtering was applied. All variants with minor allele frequency (MAF) of less than 1 % in gnomAD database, and disease-causing variants reported in HGMD®, in ClinVar or in CentoMD® were evaluated.

### 2.3 Genetic variants reported in this manuscript

Genetic variant associated with LHON: *MT-ND4* gene, homoplastic, missense, NC_012920.1:m.11778G > A/p.Arg340His, pathogenic, causally linked to LHON.^30^^,^^31^

Genetic variant associated with ADOA include: *OPA1* gene, heterozygous, nonsense, NM_130837.2:c.2383C > T/(p.Gln795∗), pathogenic, optic atrophy, autosomal dominant.^32^

Genetic variant nomenclature is in accordance with the recommendations of the American College of Medical Genetics and Genomics and the Association for Molecular Pathology.^33^

### 2.4 Case 1 (younger son)

**Fig. 1:**
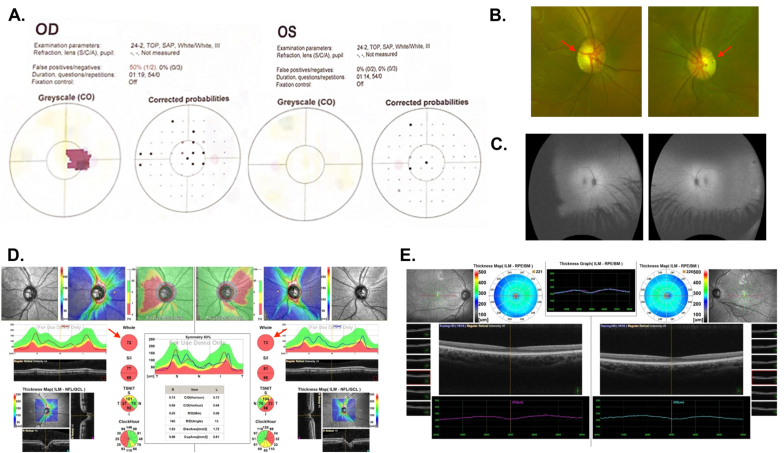
**– Case 1 (A)** Visual fields of right and left eyes. **(B)** Optos photographs of the right and left fundus showed healthy macula, normal blood vessels with optic nerve temporal rim pallor (red arrows). **(C)** Fundus auto-fluorescence showed clear media, healthy macula, normal blood vessels OU. **(D)** OCT of optic nerve of both eyes showed ONH inferior and temporal rim affection, and cup-to-disc ratio 0.6 OD, and 0.6 OS (red arrows). **(E)** OCT macula of both eyes. OCT: optical coherence tomography; OD: oculus dexter; ONH: optic nerve head; OS: oculus sinister; OU: oculus uterque. (For interpretation of the references to color in this figure legend, the reader is referred to the Web version of this article.)

A 20-year-old otherwise healthy male presented with bilateral painless decrease in vision for five years. The systemic review, ocular, medical, and drug history were unremarkable. On initial examination, the best corrected visual acuity (BCVA) was 6/30 in the right eye (oculus dexter [OD]) and 6/30 in the left eye (oculus sinister [OS]). Intraocular pressure (IOP) was 17 mmHg OD and 19 mmHg OS. The pupil was rounded, regular, and reactive (RRR), with no *afferent pupillary defect (APD) in both eyes. Extraocular movement (EOM) was full in* both eyes. Saccades and smooth pursuit were within the normal range in both eyes (oculus uterque [OU]). *Color vision was* 2/21 OD and 1/21 OS (Fig. 1A). No nystagmus was detected, and cranial nerves were intact. Slit lamp examination (SLE) was not significant. Dilated fundus examination showed clear media, healthy macula, and normal blood vessels OU (Fig. 1B and C**)**. Optic nerve head (ONH) had temporal rim pallor, cup-to-disc ratio was 0.6 OD and 0.6 OS (Fig. 1D and E**)**.

The genetic test reported a nonsense heterozygous likely pathogenic variant identified in the *OPA1* gene NM_130837.2:c.2383C > T/(p.Gln795∗). This finding is consistent with the genetic diagnosis of ADOA type 1. A missense homoplastic pathogenic variant was identified in the *MT-ND4* gene NC_012920.1:m.11778G > A/(p.Arg340His). This finding is consistent with the genetic diagnosis of LHON with mitochondrial inheritance. Magnetic Resonance Imaging (MRI) outcomes of the brain and orbits were within normal. This young male patient had combined ADOA and LHON. He was prescribed idebenone 500 mg BID (privately purchased at the available dosage of 500 mg), and at the time of reporting this case, no vision improvement was reported.

### 2.5 Case 2 (older son)

**Fig. 2:**
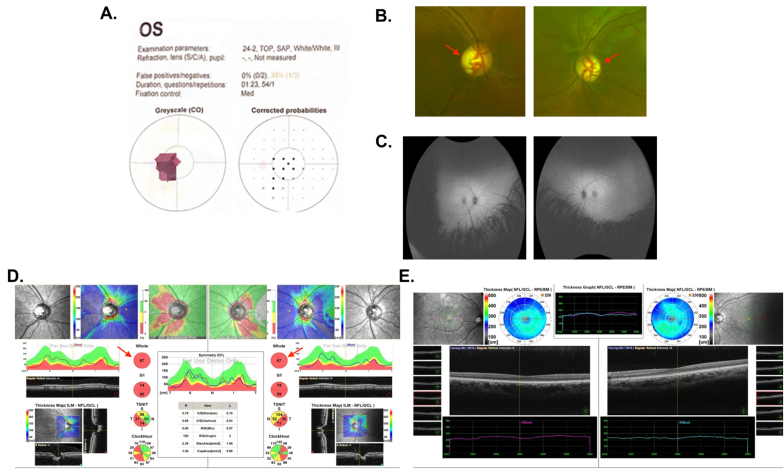
**– Case 2 (A)** Visual field of left eye. **(B)** Optos photographs of the right and left fundus showed healthy macula, normal blood vessels OU, ONH showed temporal rim pallor (red arrows). **(C)** Fundus auto-fluorescence showed clear media, healthy macula, normal blood vessels OU. **(D)** OCT of optic nerve of both eyes showed inferior and temporal rim pallor (red arrows). **(E)** OCT of macula of both eyes. OCT: optical coherence tomography; ONH: optic nerve head; OS: oculus sinister; OU: oculus uterque. (For interpretation of the references to color in this figure legend, the reader is referred to the Web version of this article.)

A 23-year-old otherwise healthy male presented with bilateral painless visual loss since he was four years old, detected on pre-school screening. He had no associated symptoms. The systemic review of ocular, medical, and drug history was unremarkable. On initial examination, BCVA 6/120 OD and 6/120 OS. His IOP was 19 mmHg OD and 18 mmHg OS. The pupil was RRR with no APD. EOM was full. Saccades and smooth pursuit were within the normal range in both eyes. Color vision was 2/21 OD and 1/21 OS (Fig. 2A). No nystagmus was detected, and cranial nerves were intact. SLE was not significant. Dilated fundus examination showed clear media, healthy macula, and normal retinal blood vessels OU (Fig. 2B and C**)**. ONH showed temporal rim pallor, and cup-to-disc ratio was 0.6 OD, and 0.6 OS. (Fig. 2D and E**)**.

The genetic test reported a nonsense heterozygous pathogenic variant identified in the *OPA1* gene NM_130837.2:c.2383C > T/(p.Gln795∗). A missense homoplastic pathogenic variant was identified in the *MT-ND4* gene NC_012920.1:m.11778G > A/(p.Arg340His). MRI outcomes of the brain and orbits were within normal. This young male patient had combined ADOA and LHON. He was prescribed idebenone 500 mg BID (privately purchased at the available dosage of 500 mg), and at the time of reporting this case, no vision improvement was reported.

### 2.6 Case 3 (father)

**Fig. 3:**
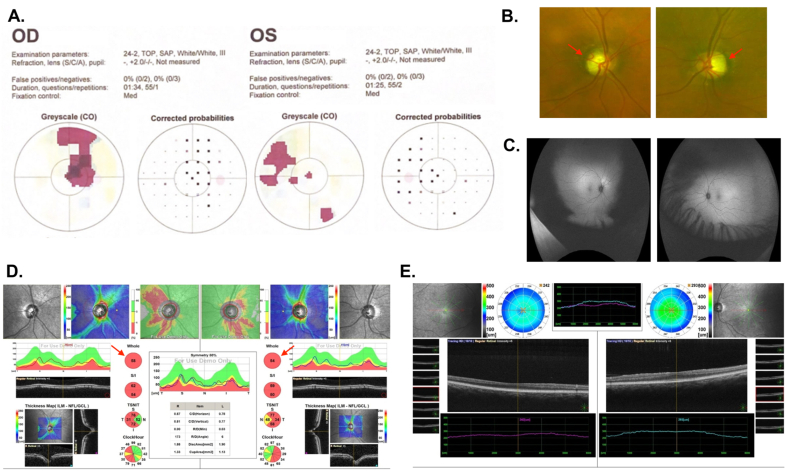
**– Case 3 (A)** Visual fields of left and right eyes. **(B)** Optos photographs of the right and left fundus showed healthy macula, normal blood vessels with optic nerve temporal pallor (red arrows). **(C)** Fundus auto-fluorescence showed clear media, healthy macula, normal blood vessels OU. **(D)** OCT of optic nerve of both eyes showed almost generalized rim thinning (red arrows). **(E)** OCT of macula of both eyes. OCT: optical coherence tomography; OD: oculus dexter; ONH: optic nerve head; OS: oculus sinister; OU: oculus uterque. (For interpretation of the references to color in this figure legend, the reader is referred to the Web version of this article.)

A 54-year-old otherwise healthy male presented with bilateral painless visual loss since childhood and no associated symptoms. The systemic review of ocular, medical, and drug history was unremarkable. On initial examination, BCVA was 6/120 in OD and 6/60 OS. His IOP was 18 mmHg OD and 18 mmHg OS. The pupil RRR no APD. EOM was full. Saccades and smooth pursuit were within the normal range in both eyes. Color vision was 1/21 OD and 1/21 OS (Fig. 3A). No nystagmus was detected, and cranial nerves were intact. SLE was not significant. Dilated fundus examination showed clear media, healthy macula, and normal blood vessels OU (Fig. 3B and C**)**. ONH showed temporal pallor, deep excavated cup, and the cup-to-disc ratio was 0.8 OD and 0.7 OS (Fig. 3D and E**)**. A nonsense heterozygous pathogenic variant was identified in the *OPA1* gene NM_130837.2:c.2383C > T/(p.Gln795∗), consistent with an ADOA diagnosis. Heterozygotes have a 50 % risk of transmitting the variant to each offspring. MRI outcomes of the brain and orbits were within normal.

### 2.7 Case 4 (mother)

**Fig. 4:**
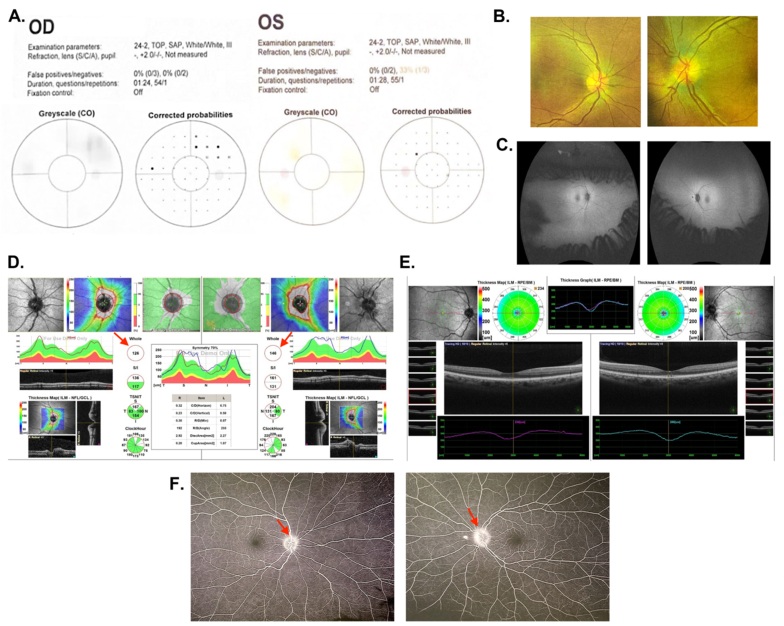
**– Case 4 (A)** Visual fields of left and right eye. **(B)** Optos photographs of the right and left fundus showed healthy macula, and telangiectatic disc blood vessels OS > OD. **(C)** Fundus auto-fluorescence showed healthy macula, and telangiectatic disc blood vessels OS > OD. **(D)** OCT of optic nerve of both eyes showed thickening in the retinal nerve fiber layer in both eyes (red arrows). **(E)** OCT of macula of both eyes. **(F)** Fluorescein angiography showing leakage in both eyes, more pronounced in OS (red arrows). OCT: optical coherence tomography; OD: oculus dexter; ONH: optic nerve head; OS: oculus sinister. (For interpretation of the references to color in this figure legend, the reader is referred to the Web version of this article.)

A 45-year-old female previously not known to have any medical illness, presented with left visual loss since she was 18 years old, secondary to solar retinopathy. She complained of headaches and flashes of light. The systemic review of ocular, medical, and drug history was unremarkable. On initial examination, BCVA was 6/9 OD and 6/12 OS. Her IOP was 22 mmHg OD and 17 mmHg OS. The pupil was RRR with no APD. EOM was full. Saccades and smooth pursuit were within the normal range in both eyes. Color vision was 21/21 OD and 21/21 OS (Fig. 4A). No nystagmus was detected, and cranial nerves were intact. SLE was not significant. Dilated fundus examination showed clear media, healthy macula, and tortuous blood vessels OS > OD (Fig. 4B and C**)**. ONH showed blurry disc margins, nasally OU, cup-to-disc ratio of 0.3, OD Patton lines, and disc vessel telangiectasia. Auto-refraction was −1.50-0.5 × 85 (OD) and −1.00-0.75 × 85 (OS) (Fig. 4D and E**)**. A missense homoplastic pathogenic variant was identified in the *MT-ND4* gene NC_012920.1:m.11778G > A/p.Arg340His. This finding is consistent with the genetic diagnosis of LHON with mitochondrial inheritance.

She also underwent brain MRI that showed signs of elevated intracranial pressure, and the lumbar puncture opening pressure was 20 cm H_2_O with normal constituents. She did not meet the modified Dandy's criteria for Idiopathic Intracranial Hypertension (IIH).^34^ Nevertheless, because of other factors like headaches, body mass index of 26.3 kg/m^2^ and brain MRI findings, she was started on acetazolamide 250 mg 4 times a day, which resolved both her headaches and optic disc swelling after a 6-month follow-up examination. Additionally, fundus fluorescein angiography showed late-stage disc leakage, which is atypical for LHON (Fig. 4F).

### 2.8 Case 5 (younger daughter)

The 15-year-old asymptomatic and otherwise healthy female was examined. The systemic review of ocular, medical, and drug history was unremarkable. On initial examination, BCVA was 6/6 OU and IOP was 19 mmHg in OU. The pupil was RRR with no APD. EOM was full. Saccades and smooth pursuit were within the normal range in both eyes. Color vision was 21/21 OU. No nystagmus was detected, and cranial nerves were intact. SLE was not significant. Dilated fundus examination showed clear media, healthy macula, and normal blood vessels in OU. ONH had a healthy rim with clear disc margins and a cup-to-disc ratio of 0.1 in OU. Auto-refraction was +0.75–1.50 × 175 (OD) and +0.75–1.00 × 9 (OS).

### 2.9 Case 6 (older daughter)

The 26-year-old asymptomatic and otherwise healthy female was examined. The systemic review of ocular, medical, and drug history was unremarkable. On initial examination, BCVA was 6/12 (OD) and 6/20 (OS). IOP was 19 mmHg in OU. Color vision was 5/21 OU. The pupil was RRR with no APD. EOM was full. Saccades and smooth pursuit were within the normal range in both eyes. No nystagmus was detected, and cranial nerves were intact. SLE was not significant. Dilated fundus examination showed a flat macula. She had a cup-to-disc ratio of 0.6 with an excavated cup, and temporal optic disc pallor in OU. A clinical diagnosis of ADOA was hypothesized given the absence of patient-reported symptoms of vision loss, especially on the background of poor prognosis associated with the LHON 11778 variant. Of note, genetic testing was denied by the family.

Table 1 provides demographic characteristics, eye examination results, and genetic test results of all six cases.

**Table 1:** – Summary of the studied cases.

Variable	Case 1 (younger son)	Case 2 (older son)	Case 3 (father)	Case 4 (mother)	Case 5 (younger daughter)	Case 6 (older daughter)
**Age (years)**	20	23	54	45	15	26
**Sex**	Male	Male	Male	Female	Female	Female
**Visual loss**	Bilateral	Bilateral	Bilateral	Left	None	None
**Visual quality**	6/30 OD 6/30 OS	6/120 OD 6/120 OS	6/120 OD 6/60 OS	6/9 OD 6/12 OS	6/6 OD 6/6 OS	6/12 OD 6/20 OS
**IOP (mmHg)**	17/19	19/18	18/18	22/17	19/19	19/19
**Pupil**	RRR No APD in OU	RRR No APD in OU	RRR No APD in OU	RRR No APD in OU	RRR No APD in OU	RRR No APD in OU
**EOM**	Full in OU	Full in OU	Full in OU	Full in OU	Full in OU	Full in OU
**Saccades and smooth pursuit**	Within normal range in OU	Within normal range in OU	Within normal range in OU	Within normal range in OU	Within normal range in OU	Within normal range in OU
**Color vision**	2/21 OD 1/21 OS	2/21 OD 1/21 OS	1/21 OD 1/21 OS	21/21 OD 21/21 OS	21/21 OD 21/21 OS	5/21 OD 5/21 OS
**SLE**	Not significant	Not significant	Not significant	Not significant	Not significant	Not significant
**Diluted fundus examination**	Clear media, healthy macula, normal blood vessels OU	Clear media, healthy macula, normal blood vessels OU	Clear media, healthy macula, normal blood vessels OU	Clear media, healthy macula, and tortuous blood vessels OS > OD	Clear media, healthy macula, and blood vessels OU	Clear media, healthy macula, and blood vessels OU
**Optic nerve**	Temporal rim pallor, cup-to-disc ratio 0.6 OD and 0.6 OS	Temporal rim pallor, disc vessel telangiectasia, cup-to-disc ratio 0.6 OD and 0.6 OS	Temporal pale, deep excavated cup, cup-to-disc ratio 0.8 OD and 0.7 OS	Blurry disc margins nasally OU, cup-to-disc ratio 0.3 OD Patton lines	Healthy rim with clear disc margins, cup-to-disc ratio 0.1 in OU	Excavated cup, and temporal optic disc pallor cup-to-disc ratio 0.6 in OU.
**Auto-refraction**	−1.00 -0.25 × 135 OD and −0.5 -0.5 × 31 OS	+0.5–0.5 × 5 OD and +0.75–0.5 × 16 OS	+1.25–0.75 × 127 OD and +1.00–0.25 × 67 OS	−1.50 -0.5 × 85 OD and −1.00 −0.75 × 85 OS	+0.75–1.50 × 175 OD and +0.75–1.00 × 9 OS	Data not available
**Affected gene/Variant type/Zygosity/Variant site/Changed amino acid**	*MT-ND4*/Missense/Homoplastic/NC_012920.1:m.11778G > A/(p.Arg340His) *OPA1*/Heterozygous/Nonsense/NM_130837.2:c.2383C > T/(p.Gln795∗)	*MT-ND4*/Missense/Homoplastic/NC_012920.1:m.11778G > A/(p.Arg340His) *OPA1*/Heterozygous/Nonsense/NM_130837.2:c.2383C > T/(p.Gln795∗)	*OPA1*/Heterozygous/Nonsense/NM_130837.2:c.2383C > T/(p.Gln795∗)	*MT-ND4*/Missense/Homoplastic/NC_012920.1:m.11778G > A/(p.Arg340His)	No genetic testing performed	No genetic testing performed

APD: afferent pupillary defect; Arg: arginine; EOM: extraocular movement; Gly: glycine; His: histidine; IOP: intraocular pressure; MT-NC: accession prefix, referring to complete genomic molecule, usually reference assembly; ND4: mitochondrial nicotinamide adenine dinucleotide hydrogen (NADH) dehydrogenase; NM: accession prefix, referring to protein-coding transcripts; OD: oculus dexter; OPA: optic atrophy; OS: oculus sinister; OU: oculus uterque; RRR: rounded, regular, and reactive pupil; SLE: slit lamp examination.

**Fig. 5:**
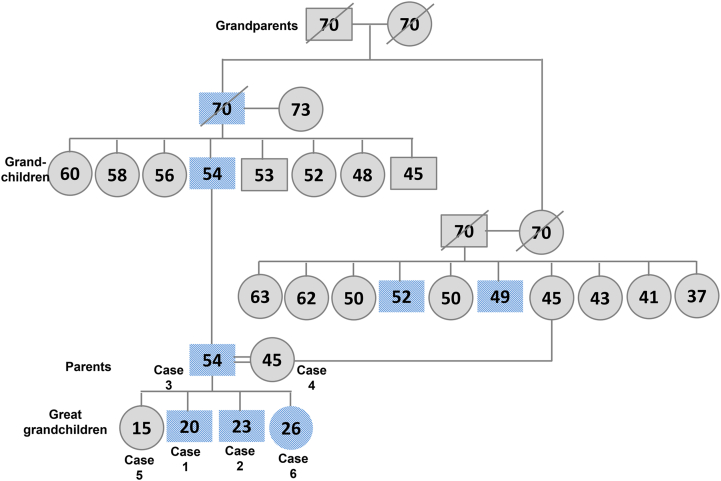
**– Family Pedigree**. Blue shaded shapes indicate affected individuals. (For interpretation of the references to color in this figure legend, the reader is referred to the Web version of this article.)

The family pedigree (Fig. 5) shows that the first generation (grandparents) were unaffected. The second generation showed an affected man who had eight children (the third generation or grandchildren); of whom one affected men (Case 3) was diagnosed with symptomatic ADOA due to the *OPA1*/Heterozygous/Nonsense/NM_130837.2:c.2383C > T/(p.Gln795∗) variant. This affected man married an asymptomatic woman (Case 4) who harbored the *MT-ND4*/Missense/Homoplastic/NC_012920.1:m.11778G > A/(p.Arg340His) variant. Their offspring (great-grandchildren) were two females: one unaffected (Case 5) and one affected (Case 6), and two affected males (Cases 1 and 2) harboring two coexisting genetic variants inherited from their parents.

## 3 Discussion

This article reports the simultaneous manifestation of LHON and ADOA due to genetic variants in two brothers, who have inherited LHON from their asymptomatic carrier mother and ADOA from their symptomatic father. LHON and ADOA are two ophthalmic disorders with distinct genetic variants despite sharing comparable potential pathways. The major three mtDNA variants in LHON are m.3460G > A, m.11778G > A, and m.14484T > C^35^; in addition to the nuclear *DNAJC30* gene recently linked to recessive LHON.^36^ A significant fraction of ADOA is caused by variants in the *OPA1* nuclear gene, which is required for mitochondrial fusion and accounts for 50–75 % of ADOA.^37^

All three LHON cases presented here had a missense homoplastic variant at position m.11778G > A. These three cases were the two symptomatic brothers and their asymptomatic mother. Over generations, families with LHON tend to have homoplastic variants, but penetrance might still vary.^12^ Environmental factors, including toxic and nutritional combination of factors (alcohol, tobacco, etc.), mtDNA haplotype, as well as nuclear DNA and compensatory mechanisms can also be key in developing mitochondrial optic neuropathies, such as LHON and ADOA, leading to variable or incomplete penetrance^38^^,^^39^ of the relevant variants. Indeed, factors such as heavy alcohol and tobacco use may precipitate disease appearance or alter disease progression and have been linked to the onset of vision loss in some LHON carriers.^40^ However, this was not the case for the 6 family members described here. Interestingly, some genetic variant carriers do not develop visual loss but remain ‘LHON carriers’; precisely due to differential genetic, epigenetic, and environmental traits.^39^ Genetic compensation may be at play, with the mtDNA copy number in critical tissues upregulated by a range of mechanisms yet not fully understood and somewhat controversial.^41^ This might be the situation encountered in Case 4 (the mother), who turned out to be a carrier of LHON genetic variant, which she passed onto her offspring, but was herself symptomatically unaffected. Most individuals present with LHON symptoms in their twenties and thirties (average age 22 years), and the vast majority (>95 %) of LHON carriers who lose vision will do so before the age of 50.^7^ However, 50 % of males and 80 % of females may never lose their vision.^7^ Consistently, the brothers received their definitive LHON diagnosis at the ages of 20 and 23 years. However, the younger brother (Case 1) had reported vision loss since the age of 15 years with optic disc pallor and cupping, and the older brother (Case 2) since the age of four. This younger age at visual impairment onset is not characteristic of LHON; but rather of ADOA, which these brothers had also inherited from their father; possibly contributing to younger onset of visual loss and to the development of symptoms leading up to the LHON diagnosis. However, the severity and the symptomatic vision loss in both brothers with different age of onset is atypical for an ADOA genetic variant alone, and a co-occurrence of both LHON and ADOA (where ADOA might have potentiated LHON manifestations) or a childhood onset LHON could explain this phenotype. Also, as a reference for comparison, the older sister had somewhat better visual acuity and color vision than her two younger brothers, which is usually seen with ADOA cases. However, this cannot be used as sole evidence for ADOA, as in a family with ADOA, there is variable expression of the disease with intrafamilial differences in the visual functions.^42^

Some LHON patients might have a better visual prognosis. The type of genetic variant is crucial, with the m.14484T > C variant associated with a higher rate of spontaneous visual recovery: at least 37 % compared with less than 5 % for the 11,778 variant, and 22 % for the 3460 variant.^43^ Childhood-onset LHON and younger age at onset is another positive factor, as younger patients are more likely to carry a relatively better visual prognosis.^44^ A larger optic disc may be a good prognostic factor for visual recovery in LHON patients and a protective factor against disease conversion in carriers.^45^ The brothers described in this manuscript carry the 11,778 variant, with the least favorable rate of visual recovery and visual acuity below 6/60 in most patients; exacerbated by a concurrent diagnosis of ADOA, known to lead to irreversible visual impairment.^25^ A recent case report described the co-existence of recessive LHON and auto-immune astrocytopathy, belonging to the neuromyelitis optica spectrum disorders.^46^ A recent review elaborated on the co-existence of LHON and multiple sclerosis, explaining that the co-occurrence of the two conditions is debatable and that LHON might be manifesting as symptoms of multiple sclerosis.^47^ The co-occurrence of LHON and ADOA in the patients presented here might theorize the potentiation of one condition by the other; underscored by the parallel exploration of the same treatments for LHON and ADOA.^47^ However, this remains at the hypothesis level, and more basic science research and clinical investigation are needed before any observation is robustly made.

In addition to the cases with two coexisting genetic variants (LHON and ADOA), the father (Case 3) was diagnosed only with ADOA, harboring the nonsense heterozygous variant in *OPA1* gene at position c.2383C > T/(p.Gln795∗). The critical distinction between ADOA and LHON is that the onset of ADOA symptoms is not sudden, and individuals with ADOA have a better prognosis overall. Furthermore, ADOA has a nuclear gene origin that affects mitochondrial function, whereas LHON often has a mtDNA origin. The loss of retinal nerve fibers can cause RNFL thinning, an essential clinical characteristic of ADOA.^48^^,^^49^

The youngest sister (Case 5) was asymptomatic, and a comprehensive ophthalmic assessment showed normal visual functions (including vision, color vision, visual field, and pupillary reflex) and normal optic disc appearance. The affected sister (Case 6) was asymptomatic, but she showed bilateral moderate reduction in vision with optic disc pallor and cupping, consistent with a presentation of ADOA alone. The father (Case 3), carrying the ADOA genetic variant, had severe bilateral vision loss with temporal pallor and cupping, which did not seem to have progressed, but had no further systemic manifestations. The mother (Case 4), a carrier of the 11,778 LHON variant, was also asymptomatic apart from headaches and mildly swollen optic discs, which both resolved upon acetazolamide treatment.

Early diagnosis is crucial to design proper management of the conditions and support the patient, in addition to identifying potential therapeutic options to halt the progression or improve visual potential. In the cases described here, Case 1 was the index patient, and he was diagnosed using whole-exome sequencing (WES); since his father (Case 3) had an optic neuropathy on exam and the patient had paternal and maternal history of vision problems; WES was more pertinent than mtDNA sequencing only. Sanger sequencing would have perhaps been a more straightforward method to confirm suspected pathogenic variants, but WES is warranted to identify other variants and secondary, including novel, findings.^50^, ^51^, ^52^ OCT has become a vital technique in the diagnosis and monitoring of LHON and ADOA patients. OCT studies showing increased thickness of the peri-papillary RNFL in the inferior/temporal quadrant are suggestive of LHON in the acute stages of vision loss,^24^^,^^53^ a thinner RNFL in the inferior/temporal quadrants is evident in ADOA initial stages.^54^ Of note, a thinner RNFL is seen in all quadrants in patients with chronic LHON and ADOA. Therefore, OCT is not helpful to distinguish LHON and ADOA in the chronic stage, as in the present cases; as in advanced stages, nerve atrophy has already set and OCT would not be of relevance.^24^^,^^54^

Currently, no curative therapies are approved for either LHON or ADOA. However, there is one disease-modifying drug, Idebenone, a short-chain benzoquinone related to coenzyme Q10 (ubiquinone) that can cross the blood-brain barrier and serve as a shuttle for electrons down the respiratory chain, resulting in adenosine triphosphate (ATP) synthesis by the mitochondrial ATP synthase in RGCs.^9^^,^^55^ This medication was approved in 2007 in the European Union by the European Medicines Agency and designated as orphan drug and in 2008 for use in Canada for LHON.^56^ A 2011 study reported that Idebenone successfully improved visual acuity in patients with LHON, over a 24-week treatment period.^57^ A 2020 study showed that Idebenone holds the potential to promote visual acuity stabilization and possibly recovery in patients with ADOA.^58^^,^^59^ Idebenone is the first, and currently only, disease-specific treatment for LHON.^55^^,^^60^ While treatment is most beneficial when started in the acute phase “within 1 year of the later eye's involvement”, clinical improvement was reported in patients who have had LHON for longer than 5 years and up to 50 years since vision loss.^61^ More recently, increased clinically relevant benefit mainly in terms of clinically relevant recovery, where improvement from “off-chart” vision to at least 1.6 logMAR, or a ≥0.2 logMAR improvement if already on-chart vision, was observed in 32.9 % of treated eyes *versus* 19.0 % of non-treated eye.^55^^,^^62^ Nonetheless, clinical management of LHON and ADOA remains challenging, and more studies are needed and specific patient profiles need to be described to predict potential benefits of starting Idebenone treatment at the chronic stage of LHON; the theory is that perhaps some of the RGCs are comatose and might still respond to treatment.^61^^,^^63^

Gene therapy is also an emerging promising option for the treatment of both ADOA and LHON, but it is not publicly available outside the experimental laboratory or clinical trial setting.^64^^,^^65^

This case series has some limitations. First, the lack of muscle biopsies or target tissues to identify the gene behind the phenotype. Second, the study is missing early medical records and tests. The lack of previous exam documentation is a major limitation, as LHON presents initially with normal or swollen optic disc that develops into atrophy while ADOA affects the optic disc with progressive pallor and cupping.

## 4 Conclusion

Mitochondrial optic neuropathies share a common pathway of mitochondrial malfunction and loss of RGCs. They cause substantial visual impairment in young individuals with no current cure. We present cases of coupled LHON and ADOA genetic variants and the challenge in distinguishing which genotype is causing the phenotype. It is unusual to find co-existing LHON and ADOA; which made the cases' definitive diagnosis challenging and the patients’ chances at a favorable prognosis less likely. Identifying such cases will help understand these rare diseases, direct patient counselling and steer the future development of new therapeutic strategies.

## CRediT authorship contribution statement

**Mohammed A. Halawani:** Writing – review & editing, Data curation. **Nooran O. Badeeb:** Writing – review & editing, Writing – original draft, Validation, Supervision, Funding acquisition, Conceptualization.

## Authorship

All authors attest that they meet the current ICMJE criteria for Authorship.

## Funding

Editing and article processing charges were provided by Biologix.

## Declaration of competing interest

The authors declare that they have no known competing financial interests or personal relationships that could have appeared to influence the work reported in this paper.
